# Emerging Adulthood, a Pre-adult Life-History Stage

**DOI:** 10.3389/fendo.2019.00918

**Published:** 2020-01-14

**Authors:** Ze′ev Hochberg, Melvin Konner

**Affiliations:** ^1^Faculty of Medicine, Technion Israel Institute of Technology, Haifa, Israel; ^2^Program in Neuroscience and Behavioral Biology, Emory University, Atlanta, GA, United States

**Keywords:** life history, adolescence, human evolution, hominin, comparative development, brain development

## Abstract

The duration of human maturation has been underestimated; an additional 4–6-year pre-adult period of “emerging adulthood,” should be included in models of human maturation. It is a period of brain maturation, learning about intimacy and mutual support, intensification of pre-existing friendships, family-oriented socialization, and the attainment of those social skills that are needed for mating and reproduction. We propose that emerging adulthood is a life-history stage that is a foundation of the high reproductive success of human beings. The period of emerging adulthood has an evolutionary context and developmental markers, and we present evidence that supports the idea that emerging adults require protection because they are still learning and maturing.

## Introduction

Growing evidence suggests that an individual at the end of adolescence cannot be considered to be an adult when using physical, physiological, intellectual, social, emotional, and behavioral measures. When adolescents in developed societies mature and achieve adult body size, their behavior often remains immature. Specialists in adolescent medicine have recognized this incongruity, and have redefined adolescence to include young adults up to age 24 years, of whom many have not yet assumed adult roles (1, 2). Reproduction in contemporary forager societies also begins several years after adolescence and post-adolescent individuals are often limited in their gathering and/or hunting skills (3–5). Compared to other mammals, primates produce few offspring. Humans have an even slower growth rate than that of non-human primates of comparable size, but human growth may be even more prolonged than is generally realized.

Arnett proposed emerging adulthood as a phase of life between adolescence and full-fledged adulthood, with distinctive demographic, social, and subjective psychological features (6, 7). This life- history stage applies to individuals aged between 18 and 25 years, the period during which they become more economically independent by training and/or education. Previously, the psychodynamic theorist Erik Erikson identified a stage that he called a *prolonged adolescence* or *psychosocial moratorium* in young people in developed societies (8, 9). Much more recently, Hopwood and colleagues explored genetic and environmental influences on personality development during the transition to adulthood in same-sex male and female monozygotic and dizygotic twins assessed in late adolescence (approximately age 17 years), emerging adulthood (~24 years), and young adulthood (~29 years) (10). Their genetically-informed results support a life-course perspective on personality development during the transition to adulthood. In addition, the United Nations has identified youth, defined as the period from 15 to 24 years of age, as a period of vulnerability worldwide and has made it a priority for multiple interventions (11).

Here, we use an evolutionary approach in order to understand emerging adulthood, arguing that it is not just a sociological transition period but a biological life-history phase. Trait variability, whether it is molecular, cellular, physiological, morphological, or behavioral, is the leading edge of evolution. Together with genetic evolution, plasticity in developmental programming has evolved to provide the organism with traits that can secure its survival and reproductive success (12). Life-history theory is a powerful tool for understanding child growth and development from an evolutionary perspective (2, 13, 14). We provide evidence that emerging adulthood exists in some other mammals, which implies genetic evolution, and we discuss emerging adulthood in foraging as well as developed societies, which implies the occurrence of adaptive plasticity and cultural influences. We propose that genetic and cultural evolution have interacted to produce the emerging adulthood stage in human life history.

## Defining the Transition from Adolescence to Emerging Adulthood

Determining the exact time of transitions between life-history stages is challenging (13). Saltations (growth spurts) and transitions occur during human growth (15, 16), and stages have a central place in evolutionary life-history theory, but the turning points are theoretical constructions in which some aspects of a transition are highlighted.

Puberty produces an endocrine transformation with striking somatic and behavioral changes, especially in body image, sex identity, aggression, and impulsivity. To define a maturational stage between adolescence and adulthood, we need first to define the end of adolescence. During this transition, growth velocity decelerates, blood and tissue hormone levels increase, aggression becomes less overt, and learning and maturation mitigate hormonal impact.

Using maturational measures avoids the pitfalls of defining emerging adulthood according to chronological age. For example, the Tanner scale of adolescent development is based on external primary and secondary sex characteristics. Tanner stage V recognizes the conclusion of puberty in boys when the testicular volume is >20 ml and the length of the penis is >14 cm (17) and in girls when the breast reaches final adult size and the areola returns to the contour of the surrounding breast with a projecting central papilla (18).

Here, we define the transition between adolescence and emerging adulthood as occurring when growth returns to its prepubertal trajectory and the boy or girl is at Tanner stage IV (19). Boys at this stage have a testicular volume between 12 and 20 ml, their scrotal skin darkens, and the length of their penis is ~10 cm. Girls at this stage have experienced menarche, their breasts are of adult size and elevated, and the areola and papilla form a secondary mound which projects from the contour of the surrounding breast. Body composition continues to change during emerging adulthood, in terms of relative fat mass, lean body mass, and total body bone mineral content and bone mineral density increase (20), but the most important maturational changes after adolescence, even if defined as the end of Tanner Stage 4, are in the brain.

Brain size may be a pacemaker in mammalian life history (21), and it underlies the remarkable human capacity for learning and communication, but the length of the brain's developmental trajectory was until recently underestimated. It is now clear that brain development does not stop with the completion of puberty when adult brain *size* is attained. Brain maturation continues beyond adolescence, extending until around age 25 years, and this recently discovered prolongation provides critical support for emerging adulthood as a post-adolescent maturational stage (22). Compared to other primates, human newborns are neurologically and behaviorally altricial because many aspects of brain development are protracted, including that of the prefrontal cortex (23). The cortical architectural units or minicolumns in the prefrontal cortex of humans are wider than those of the great apes, an increase that occurs after puberty in humans, but not in chimpanzees (24). In chimpanzees, but not in humans, myelination becomes complete at about the time of sexual maturity (25). Interestingly, human brain regions with protracted development are the same that have undergone the greatest degree of volumetric enlargement in primate evolution (26).

In a large-scale longitudinal pediatric neuroimaging study, brain maturation was found to continue after adolescence: post-adolescent increases in white matter are linear while the changes in the cortical gray matter are non-linear. Cortical white matter in particular continues to increase into the mid-twenties, which is likely related to the efficiency and speed of cortical connectivity (27, 28). In another study, Sowell and her colleagues spatially and temporally mapped brain maturation in North American adolescents (age 12–16 years) and young adults (age 23–30 years) using a whole-brain, voxel-by-voxel statistical analysis of high-resolution structural magnetic resonance images (29). They found that the pattern of brain maturation during these years was distinct from earlier development and was localized to large regions of the dorsal, medial, and orbital frontal cortex and lenticular nuclei. They also reported relatively little change at other brain locations. They concluded that cognitive function improves throughout adolescence, and this improvement is associated with parallel post-adolescent reductions in gray matter density (as white matter increases) in frontal and striatal regions. It has been argued that such brain changes should mitigate the guilt of adolescent delinquents who have not yet gone through them (30–32).

Asato and colleagues also investigated white matter maturation during adolescence using diffusion tensor imaging and reported that (a) pubertal hormones influence white matter development and maturation and (b) white matter connectivity and the executive control of behavior is still immature in adolescence (33). Jolles and colleagues investigated the association between whole-brain functional connectivity and cognitive and emotional functions in children (11–13 years) and young adults (19–25 years) (34). Although they found similar patterns of functional connectivity in children and young adults, there were differences in the size of the functionally connected regions and the strength of functional connectivity. Thus, functional connectivity continues to change during and after adolescence, and these developmental differences in functional connectivity patterns were associated with higher cognitive or emotional functions and basic visual and sensorimotor functions.

In another study comparing social and emotional functioning of children, adolescents, and young adults, by analyzing the age-dependent development of five functionally distinct cingulate-based intrinsic connectivity networks (ICNs), Kelly and colleagues provide additional evidence that brain maturation extends beyond adolescence into young adulthood (35). They found that the pattern of correlation with voxels proximal to the seed region of interest was age-dependent: the pattern was diffuse in children (mean age 10.6 years), was less diffuse in adolescents (mean age 15.4 years), and showed signs of becoming focal in young adults (mean age 22.4 years). Also, the greatest development occurred in those ICNs associated with social and emotional functions. Finally, in their study of the brains of 103 healthy subjects aged 5–32 years using diffusion tensor tractography, Lebel and Beaulieu provide further evidence that brain maturation continues from childhood into adulthood (36). Association tracts show within-subject maturation of measures indicative of myelination and axon density.

Collectively, these studies provide strong evidence that brain development and maturation continue in young adulthood; the idea that brain maturation is finalized during adolescence is no longer tenable. Psychologically, emerging adulthood is a stage when an individual's cognitive abilities increase to reach their peak in their fourth decade and possibly beyond (37). Schaie and colleagues included 13 measures of crystallized abilities influenced by schooling and experience. The critical abilities from this perspective are those that enable the learning of new things, that is, working memory and fluid intelligence; these, as well as processing speed (38), peak in the mid 20s.

Emerging adulthood is also a social stage: it is a period of learning about intimacy and mutual support, intensification of pre-existing friendships, family-oriented socialization, political awareness, developing new relationships, and the attainment of biosocial skills that are needed for successful mating and reproduction. Finally, it is also a stage of understanding self-concepts and ideal concepts, emphasized interpersonal reactivity and obligation, self-expressiveness, and contempt toward particular ideologies (39). The attainment of these cognitive, emotional, and social abilities is the result of a complex interplay of maturation and interaction with the environment, but it is now possible to say that at least in the earlier years of emerging adulthood, they are correlated with and possibly caused by brain maturation. There is also evidence that brain size growth continues into the third decade in some individuals. In these individuals, hypothalamic maturation, puberty, and the resultant hormonal surges are dissociated from and even precede development and maturity of frontal cortex (40, 41).

**Figure 1:**
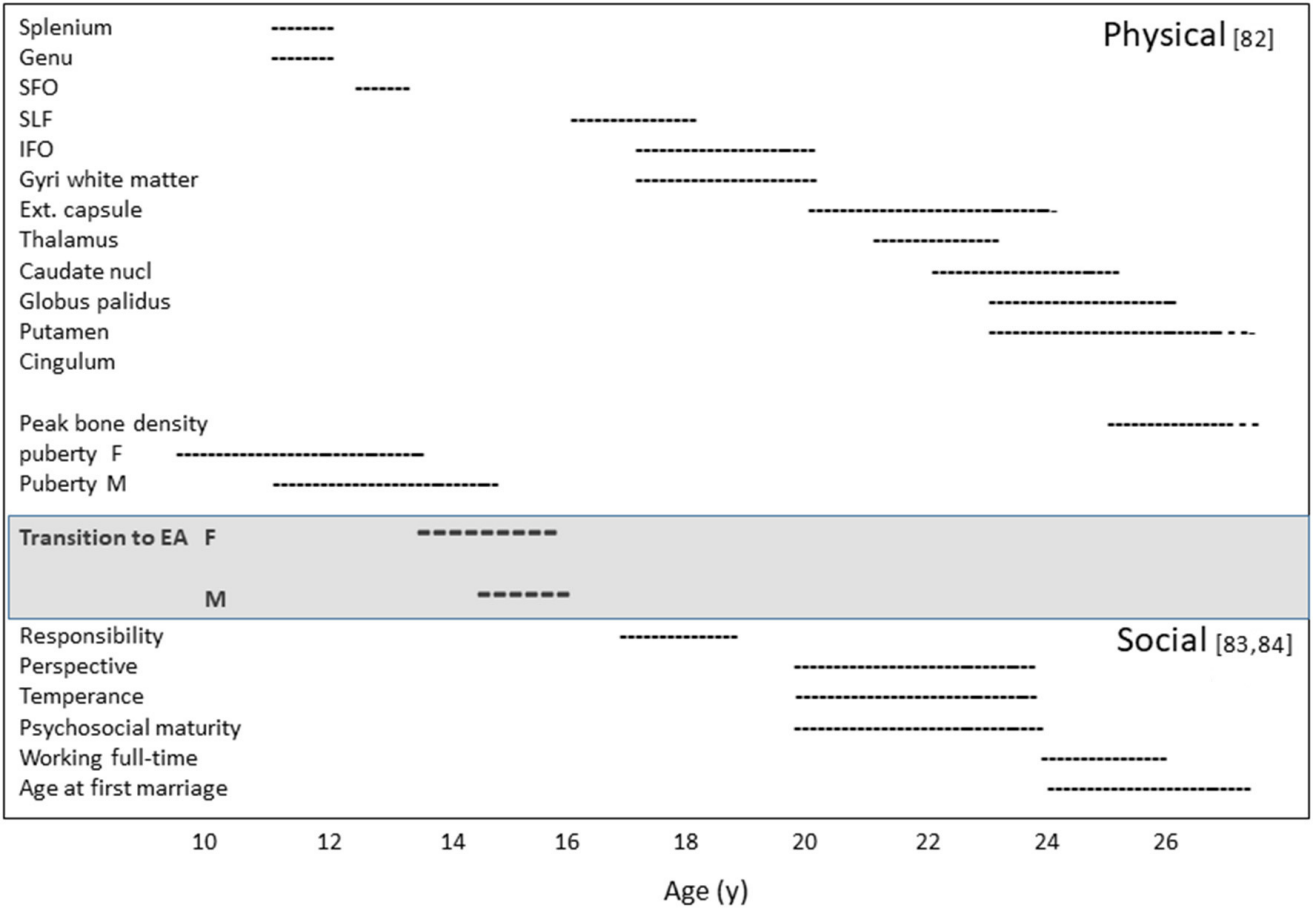
Maturation timeline: (Upper) the age range to complete physical maturation (82). (Lower) the age range to complete social maturation (83) and US Bureau of Labor Statistics, 2014. SFO, The subfornical organ; SLF, The superior longitudinal fasciculus; IFO, anterior insula/frontal operculum complex; EA, emerging adulthood; F, female; M, male (82–84).

Emerging adulthood is associated with other physiological changes, such as bone mineral accretion, the completion of growth, and [frequently] first reproduction. Hence, emerging adulthood begins as a physiological, but most importantly a neural transformation in which behavioral and social functions interact, with consequences for impulse control in domains that have put the individual at risk during puberty. We will argue that this life-history phase has unfolded throughout hominin evolution. In Figure 1 we show the timeline of maturation of the main physical, behavioral and social traits.

## Growth-related Definition of the Transition to Emerging Adulthood

**Figure 2:**
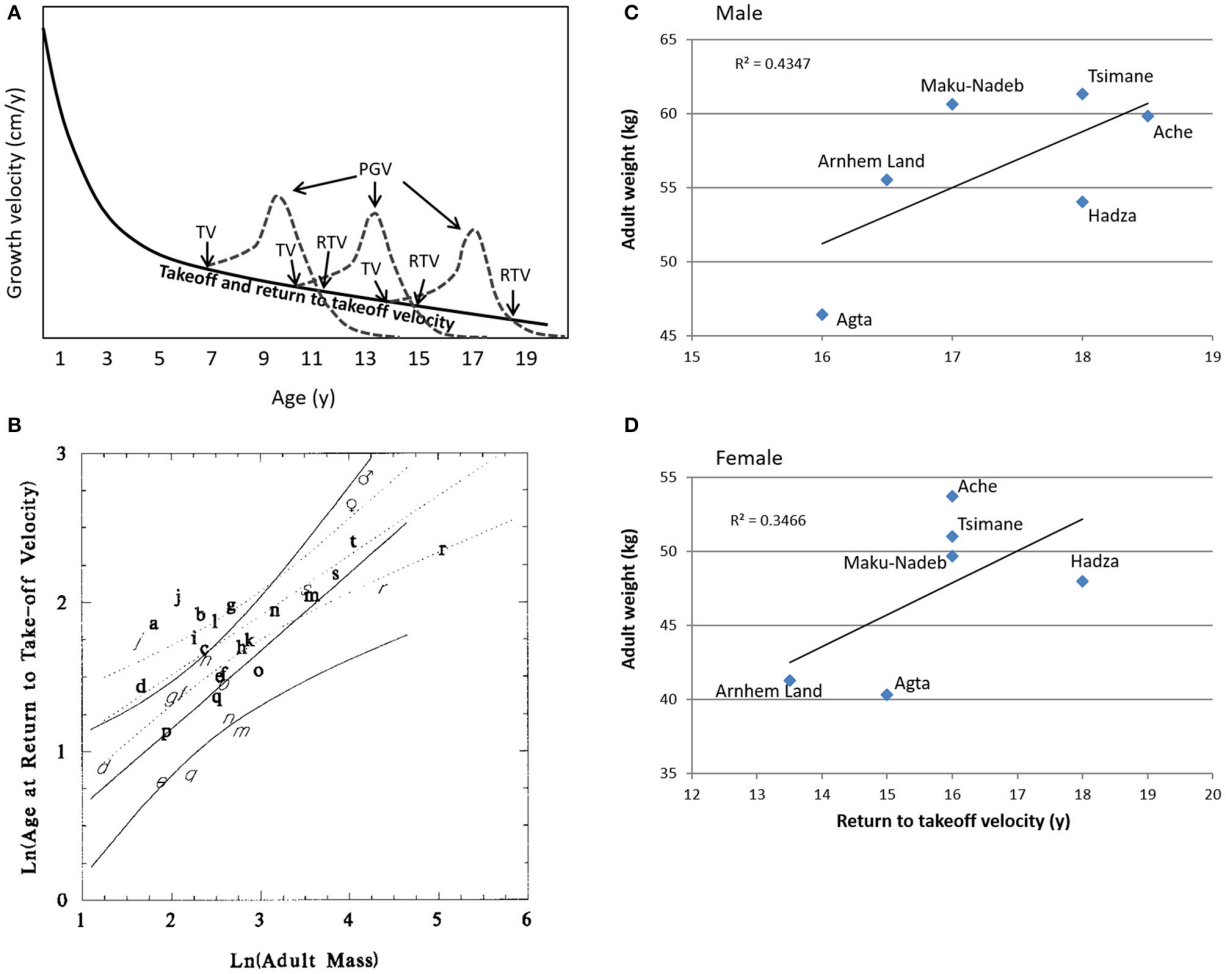
The concept of the return to prepubertal growth velocity curve as a life history mile stone. Here, we define the return to take-off velocity as the transition from adolescence to emerging adulthood. **(A)** Schematic representation of the age-dependent pubertal take-off velocity and the return to prepubertal growth velocity curve; in girls the return to takeoff velocity coincides with menarcheal age in the early, average, and late maturers. The age-dependent decline in peak height velocity is a function of the decelerating takeoff velocity and returns to the prepubertal growth velocity curve. PGV, peak growth velocity; TV, takeoff velocity; RTV, return to prepubertal growth velocity curve. **(B)** The age of return to prepubertal growth velocity curve as a function of adult body mass in 21 primate species. Observations are derived from captive primates held at zoological parks and primate centers. With permission from Leigh and Park (42). a—*Cebus apella*, b—*Cercopithecus aethiops*, c—*Cercopithecus mitis*, d—*Cercopithecus neglectus*, e—*Erythrocebus patas*, f—*Cercocebus atys*, g—*Macaca arctoides*, h—*Macaca fascicularis*, i—*Macaca fuscata*, j—*Macaca mulatta*, k—*Macaca nemestrina*, l—*Macaca silenus*, m—*Papio hamadryas*, n—*Mandrillus sphinx*, o—*Colobus guereza*, p—*Presbytis entellus*, q—*Presbytis obscura*, r—*Gorilla gorilla*, s—*Pan paniscus*, t—*Pan troglodytes*. **(C,D)**—average adult body weight as a function of the age at return to prepubertal takeoff growth velocity of males **(C)** and females **(D)** in six predeveloped societies. Data from http://dice.missouri.edu.

To define the transition from adolescence to emerging adulthood, we use the age at which growth velocity returns to prepubertal levels (Figure 2A). The adolescent growth spurt can be identified from the growth velocity curve, and its takeoff is signaled when the rate of growth changes from deceleration to acceleration at the end of the juvenile stage (13). This inflection point marks the beginning of the adolescent growth spurt. The point at which the curvilinear growth velocity spurt returns to the pre-takeoff velocity defines for us the end of adolescence and the beginning of emerging adulthood. This refinement of the “return to [pre-]takeoff velocity,” which was previously proposed by Leigh and Park (42), is essential for understanding the human pubertal growth spurt (43). This model, displayed in Figure 2A, explains the apparently diminished peak height velocity in delayed puberty and is the basis of adult height predictions for prepubertal children.

In an allometric analysis of 21 species of anthropoid primates, the age at return to pre-takeoff velocity and the adult body mass are positively correlated in both females and males (Figure 2B). The age at return to pre-takeoff velocity occurs later in human beings than other primates because of the lateness of our growth spurt when body mass is considered (42). Overall, the growth spurt in most primates is quite minimal, and little is known about the relationship between the age at return to prepubertal growth velocity and the appearance of secondary sexual characteristics at puberty. Takeoff velocity occurs early in gorillas, and despite their greater body mass, female gorillas become sexually mature at a younger age than female chimpanzees (44). Similar to humans, vervet (*Cercopithecus aethiops*) and rhesus monkeys (*Macaca mulatta*) show a relatively late return to prepubertal growth velocity. Interestingly, this positive correlation between the age at return to prepubertal growth velocity curve and body mass also exists in six small-scale societies described in Walker's Database for Indigenous Cultural Evolution (http://dice.missouri.edu/) (Figures 2C,D).

## The Evolutionary Context of Emerging Adulthood

### Evolutionary Life-History Theory

**Figure 3:**
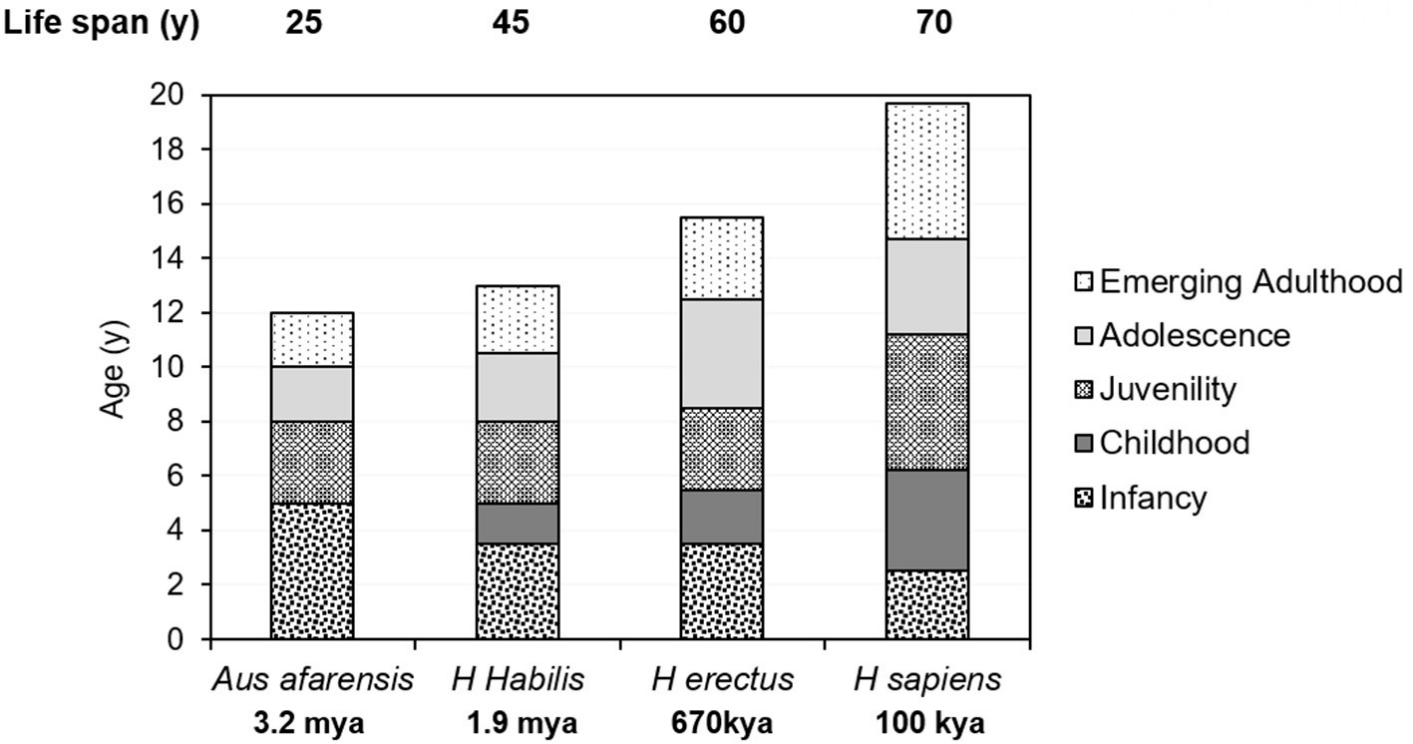
Comparison of the life-history stages and the newly defined emerging adulthood of *Australopithecus afarensis, Homo habilis, H. erectus*, and *H. sapiens*.

Life history is defined as the allocation of an organism's energy toward growth, maintenance, reproduction, raising offspring to independence, and avoiding death (45), and adaptation to environmental changes requires the selection of certain life-cycle traits (46, 47) (Figure 3). Evolutionary life-history theory attempts to explain and predict tradeoffs that optimize energy expenditure, reproductive advantage, and risk (2, 14, 48, 49). Central to the concept of sexual selection is the attainment and optimization of reproductive competence, and the key traits for selection are growth, maturation, and the age at transition to adulthood and sexual reproduction (12).

Human beings and the great apes share similar traits including, to some extent, emerging adulthood. We know relatively little about neurological maturation in non-human primates, but we do know that non-human great apes have a 2-year period of post-menarcheal infertility (50), extended in human foragers to 3 years (51). Low reproductive success among young females is a general primate phenomenon (52). Male preference for fully developed adult females has been described in 15 primate species (52). Goodall reported that after menarche, which usually occurs at age 10 years, female chimpanzees average 19 full-size cycles before becoming pregnant for the first time at age 12 years (53). Moreover, they will have about 60% of their lifetime sexual encounters during this post-menarcheal period. Unlike gorillas, chimpanzees (*Pan troglodytes)*, and bonobos (*Pan paniscus*) live in multi-male and multi-female groups and mate more often than needed to conceive. Accordingly, primatologists have suggested that adolescent sterility is a period in which sexual and social skills are practiced without responsibility for the care of a newborn (53). In their emerging adulthood, female vervet monkeys (*Cercopithecus aethiops*) display a high degree of interest in young infants and will touch, cuddle, carry, and groom infants whenever they can. Lancaster interpreted this play-mothering by young females as an opportunity to practice maternal behavior and ease into their expected maternal role in society (54). Fecundity in males depends on age, size, and experience. Similar to humans, where reproductive success is in-line with hunting ability (55) reproductive success among the Barbary macaques (*Macaca sylvanus*) is much lower in young than adult males (56). In male chimpanzees, pre-fertility copulation is very common (53).

While it seems impossible to ascertain the life-history stages of early hominids, the timing of their dental maturation from the fossil record has shed some light on their stages (see below). Australopithecines are anatomical intermediates between apes and human beings and chimpanzees and bonobos are often regarded as living species that can to some extent represent the australopithecines. Based on fossil dental specimens, australopithecined resembled wild chimpanzees, not modern humans, in life-history stages (57). Fossil *Homo* species matured more slowly and the attainment of certain developmental milestones, such as the onset of puberty, adolescence, and first reproduction, probably occurred later, in parallel with their increasing longevity, body mass, and height (Figure 3).

A life-history tradeoff is a fitness cost that occurs when a beneficial change in one trait is linked to a detrimental change in another trait (58). According to Charnov, a life-history tradeoff also entails an invariant in an underlying parameter that the life cycle stabilizes or is constrained by (59). The *Charnov model* of mammalian life-history evolution (59) derives the flow of life-history consequences from the adult mortality rate:

*Adult mortality* □ *Age at maturity* □ *Adult weight* □ *Fecundity* □ *Juvenile mortality*.

In this model, any factor that decreases adult mortality, such as large adult body mass, sociality, or a low-predation environment, favors delayed maturation. Reproductive value (RV) increases with body mass while growth rates decline. The optimal age to stop investing in growth is when the expected RV starts to decline. Body mass increases during growth, which stops when body mass is optimal, so juvenile survival becomes important when maturation is delayed. Increasing juvenile survival and extending the adolescent life-history stage increases that individual's RV. Hence, emerging adulthood is highly favored.

The offspring number of most species with a large body size is small. Additionally, juvenile mortality decreases when reproduction is late, and late reproduction is associated with high fertility. Late reproduction should decrease fitness (60), but several tradeoffs could influence the prolonged period of emerging adulthood in human life-history strategies: reproducing at an earlier or later age; reproducing at a young age or continuing to grow and develop; and being an adult parent with a large parental investment in each offspring of a small family or a young parent with a small parental investment in each offspring of a large family. The Charnov model predicts that a long life span will be associated with slow development, iteroparity (repeated reproduction), a single offspring, and long parental care (59). It was recently suggested that slow rates of growth, reproduction, and aging among primates reflect their low total energy expenditure (61). Emerging adulthood in modern societies is part of the historical lengthening of both ends of the pre-reproductive life span of human females (early puberty and late reproduction) in response to improved nutrition and decreased infection (62, 63). Microevolutionary tradeoffs that might underlie an extended emerging adulthood stage of life history include the allocation of energy to growth or reproduction, and the energy investment in courtship or parenting. Indeed, performing the sexual act in some species requires good cognitive ability and specific sexual behaviors (64). During human evolution, the acquisition of certain abilities resulted in the lengthening of maturation and development.

Brain size in mammals is correlated with longitudinal growth, and both have increased during human evolution (65). Brain size and cultural complexity have concomitantly increased over the last 2 million years with two possible periods of accelerated increase. The first occurred during the early evolution of the genus *Homo*, the second during the rise of *Homo sapiens*.

Using Charnov's model, we also suggest that emerging adulthood is a life-history stage that is a foundation of the high productivity of human beings: the metabolic potential of human beings exceeds the metabolic requirements of survival and this excess is first used to support growth and brain maturation before being allocated to reproduction. Another critical adaptation in hominin evolution was the ability to improve the food supply by establishing a rich and stable food base through the control of fire (*Homo erectus*), cooking (early *Homo sapiens* or earlier) (66, 67), and exploiting coastal food resources (shellfish) (68).

Despite the fact that the human juvenile (including emerging adulthood) and adult periods are longer than that of the chimpanzee and that human infants are larger than chimpanzee infants at birth, hunter-gatherer women characteristically have higher fertility than chimpanzee females (69). In anthropoid primates (monkeys, apes, and humans), non-maternal care predicts earlier weaning, shorter birth spacing, and higher reproductive success (70). Parental provisioning of the weaned offspring, an aspect of cooperative breeding (71), is crucial (72, 73). Here, we argue that anatomically modern human parents care for their offspring throughout their offspring's adolescence and emerging adulthood, and this extended period of care is longer than that of other primates.

The unique evolutionary path to the genus *Homo* was shaped by an increasing reliance on calorie-dense, large-package, skill-intensive food resources, “which, in turn, operated to produce the extreme intelligence, long developmental period, three-generational system of resource flows, and exceptionally long adult life characteristic of our species” (74). Kaplan and Robson emphasized the role of human males in provisioning meat to their family and tribal members (74). They also highlighted the contributions of grandmothers and other family and band members to provisioning and childcare, enhancing survival and success in emerging adulthood.

**Figure 4:**
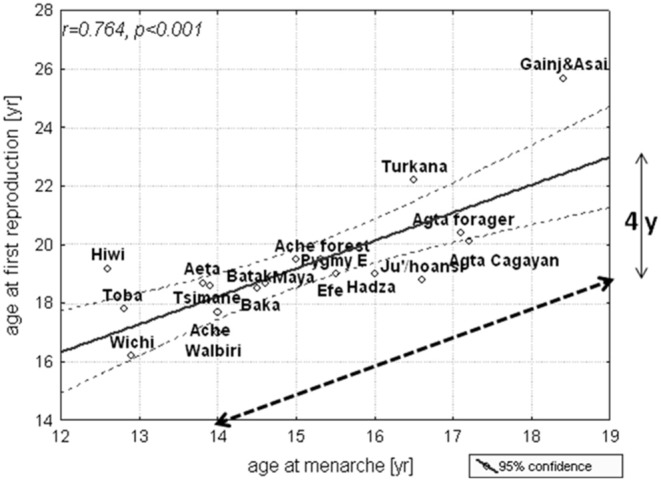
Age at menarche and first birth among some natural fertility societies; mean and 95% confidence limits. The dashed line is the age at menarche when plotted against the age at first reproduction [reproduced with permission from Hochberg et al. (76)].

Although, the average menarcheal ages of the gorilla, the bonobo, and the chimpanzee are 7–8, 9, and 11 years, respectively, their ages at first birth are 10–12, 13–15, and 14–15 years (75). Despite their rapid development compared with humans, the great apes have a distinct period of post-menarcheal. In parallel with other great apes, the menarcheal age of human forager populations ranges from 13 to 19 years, and their first birth occurs about 4 years later when they are between 17 and 23 years of age (62, 76) (Figure 4). In contrast to great apes, primiparous women of human forager populations are provisioned by mature adults, such as grandmothers, who are usually post-reproductive (77, 78); their husbands, who are typically several years older and have often passed through the emerging adulthood stage of their life history before marriage; and other adults (3, 79–81).

Despite similarities among primates, the prolongation of dependency during emerging adulthood (Figure 3) is unique to human life history, and is part of the evolutionary success of *Homo sapiens*. In the light of the knowledge we have gained from other primates, we need to improve our existing definitions of the beginning and end of emerging adulthood in primates in terms of physical traits.

### Development of the Human Reproductive Strategy

Across forager societies, there is a consistent 3–4-year period between menarche and the birth of the first child (Figure 4) and adult reproductive behaviors are learned during this period of emerging adulthood. The evolution of human development culminated in environment-dependent and late reproductive maturation. According to life-history theory (59, 85), a reduction in juvenile and adult mortality (86) postponed reproduction and necessitated substantial parental investment in each offspring.

Sexual behavior develops according to a species-specific, genetically controlled, maturational plan in which the age at first reproduction occurs within a specific age range (87). Darwinian theory yields testable predictions about mating strategies and behaviors, which include jealousy, fidelity, pursuit, diffidence, the number of new sexual partners per year (partner frequency), and gender roles and behaviors customarily displayed in emerging adulthood (88). These predictions also apply to adult patterns of intra- and intersexual aggression (89, 90). Here, we offer an evolutionary model for this transformative life-history stage, emerging adulthood.

Despite cross-cultural variations in the age of initiation of sexual activity and the age at marriage, the period of emerging adulthood in all cultures involves readiness for mating. Strong emotions often accompany early sexual activity. During adolescence, the frequency of depressive episodes is temporarily increased in boys and especially in girls (91–93).

Sex hormones intensify adolescent behavioral and psychological changes (94–96), but in emerging adulthood and into adulthood, average rates of depression, anxiety, and risk-taking decline. Interestingly, serum testosterone levels continue to rise after puberty and peak in the third decade in male humans (97). However, this age-dependent increase in serum testosterone levels does not occur in chimpanzees: serum testosterone levels are higher in adolescent chimpanzees (age 7–10 years) than in adults (age >11 years) (98). Male and female sex drive may be intensified and/or enabled by the activational effects of the sex steroids, as part of a switching mechanism that re-allocates resources from growth to reproductive activity during emerging adulthood (99). If the same genes allocate the energy that is required for growth and reproduction, these genes could exhibit antagonistic pleiotropy and mediate the tradeoff between growth and reproduction (100). The “fight or flight” response to perceived threat influences life-history tradeoffs during development (101, 102). As part of their readiness for mating, the bullying behavior of adolescent males diminishes at the time of transition to emerging adulthood (103, 104). This could be due to adolescents learning subtler ways of competing, they still vie for dominance and resource control. This may help explain why the mean age difference between men and women at the time of their first marriage in 191 national populations and traditional societies is 3.5 years (105).

### Adolescence and Emerging Adulthood Among the !Kung and Other Foragers

Contemporary forager societies are to some extent modern representatives of pre-agricultural forager societies. The !Kung were until recently foraging people of the Kalahari Desert, whose demography and life history have been extensively studied (81, 106). Their average age of menarche is 16.6 years (range 16–18 years), and about 50% were married before menarche to men averaging 10 years older. Their age at first childbirth was 19 years (range 17–22 years) (106). This 3-year period of between the age of menarche and the age at first birth is probably due to subfertile ovarian cycling. Although their husband's sexual advances were supposed to be delayed until menarche, women reported that this period was often stressful (107). This 3-year period is important for a newly married !Kung woman for at least two reasons. First, she gradually learns to adopt adult roles and acquire adult sexuality without having to deal with the consequences of pregnancy and feeding a family. Second, she usually lives near her mother, even after the first birth, because she is dependent on her mother, father, and extended family before moving to her husband's village-camp after a second child (81, 107). Although !Kung women become socially responsible mothers with two or more children by their mid-20s, they are typically still being provisioned by their families (81). Psychosocial development during emerging adulthood is substantially longer in boys than in girls, and the transition from adolescence to adulthood is gradual (108). !Kung boys learn hunting and other subsistence skills and are permitted to accompany adult men on hunting trips from their mid-teens. But the husband's obligation to provision his family with meat is also aided by relatives during the period of emerging adulthood.

To what extent do the !Kung resemble other hunter-gatherer societies? The acquisition of subsistence skills is a very long process among the closely related San of the Okavongo Delta, Botswana (5). Mongongo nuts are a staple food (as for the !Kung) and the ability to crack these nuts is age-specific because nut-cracking requires skill; arm strength is less important than age. Plotted against age, the ability follows an inverted U-shaped function across the lifespan, and this time-dependent function is a good example of the adaptive evolutionary value of emerging adulthood beyond adolescence. Success at nut-cracking is minimal until the late teens and then this skill improves until midlife.

The Hiwi Indians of Venezuela and the Aché Indians of Paraguay are traditional hunter-gatherer groups whose hunting and subsistence skills gradually increase throughout young adulthood (109). Although Aché girls collect insect larvae for subsistence, children of the two tribes under age 10 years do almost no foraging and especially no hunting until their teenage years. Specifically, the skill of gathering honey and palm fiber of Aché boys and Hiwi girls progressively increases to levels that are about half of their peak adult values in adolescence. The age at which the hunting skills of Hiwi and Aché men are at their best is the late 30s, and the age at which Hiwi and Aché men and women reach their peak gathering skills for honey and palm fiber occurs is even later.

Tsimane foragers of Bolivian Amazonia are also relevant to the long pre-adult life history of modern humans (4). Based on hunting returns and specific skill tests, the peak performance of hunters is only reached several years after the completion of a long childhood and adolescence; hunters must first learn to recognize the sounds, smells, tracks, and feces of critical prey species, and then learn to hunt by sightings, pursuits, and attempted kills. The hunting performance and ability of Tsimane foragers is another example of a skill whose acquisition depends more on age than strength.

Thus, the evidence from foraging societies and the conditions to which humans became adapted during our evolution show that neither reproductive behaviors (i.e., parenting and the ability to manage the relationship with a spouse) nor subsistence skills are mastered by the end of adolescence. Even in societies where children forage from an age as young as four, their efficiency as young adults remains lower than that of their mothers (110). Blurton Jones and Marlowe confirmed increases in skill and performance with age in the Hadza, hunter-gatherers of northern Tanzania. For example, the accuracy when shooting with a bow and arrow among men Hadza people increases with age and reaches its peak at age 25 years (111). From such findings, Blurton Jones and Marlowe concluded that one cannot assume that the age-dependent increase in performance and ability is entirely due to learning and/or practice; the increase may also be due to increases in an individual's size and strength (111). The importance of size and strength is confirmed by a study of spearfishing and shellfishing efficiency among the Meriam, who live on the Mer and Dauer islands in the eastern Torres Strait. For fishing and spearfishing, which are cognitively difficult, Bird and Bird found no significant amount of variability in return rates because experiential factors correlated with age. However, for shellfish collecting, which is relatively easy to learn, they found strong age-related effects on efficiency (112). From the evidence collected from various foraging societies around the globe, performance proficiency of subsistence skills of individuals increases with age and only peaks when they transit from emerging adulthood into adulthood in their twenties or later. These findings confirm that the period of emerging adulthood is marked by age-dependent maturation, ongoing brain development, strength accrual, and learning, and is a key adaptation for human survival and reproduction.

## Secular Trends In Adolescence and Emerging Adulthood

Menarcheal age has declined in the U.S. and Europe for over a century (113, 114). It has declined by 4 years over the past 150 years, and the age at peak height velocity in the pubertal growth spurt has also decreased by 4 months per decade (114). An evolutionary approach to this secular trend challenges the concept that early adolescence is a disease process, and suggests that contemporary reproductive and life-history strategies are reflected in the substantial increase in the presentation of females with early-onset adolescence (115–118). Part of the misconception that early adolescence is a pathological condition is related to the assumption that the transition from adolescence to adulthood is direct. The subfertility of emerging adulthood can be explained by the period between the age at menarche, which is 12.5 years, and the modeled optimal age at first birth of 18 years (119). Indeed, puberty is followed by subfertility in adolescence and emerging adulthood (120) due to a high proportion of non-ovulatory cycles (121). Currently, there is no evidence for a secular trend in the age at first consistent ovulation.

Despite liberal mores and adolescent sexual activity, early childbearing was uncommon in pre-agricultural societies. In a non-industrial traditional society, a girl who begins to menstruate at age 15 years can take her place in that society at age 19 years as a young mother after a 4-year period of emerging adulthood and be supported by the institutions of marriage and an extended family (Figure 4) (76). In developed societies, the period of emerging adulthood of a girl who begins to menstruate at 12 years is prolonged, with slow maturation of the prefrontal cortex and other brain structures and late myelination until at least age 25, producing the mismatch between early-onset of puberty and late mental maturation in contemporary developed societies (116). It is the later part of this period of mismatch that we define as emerging adulthood, a time when young adults are still immature in their judgment and incapable of performing adult tasks (82).

## Summary and Conclusions

The idea that one of the outcomes of human evolution is a very prolonged period of adolescent growth and delayed maturity is old, and is consistent with life-history theory, comparative primatology, and the hominin fossil record. We suggest in addition that emerging adulthood is a life-history stage that is part of the foundation the high productivity of human beings: our metabolic potential exceeds the metabolic requirements of survival and this excess is first used to support growth and brain maturation before being allocated to reproduction. We contend that the duration of human maturation has been underestimated, and that an additional 4–6-year pre-adult period, which (following Arnett) we call emerging adulthood, should be included in human life history. Recent imaging studies have shown that brain development continues throughout emerging adulthood; maturation of the neocortical association areas, notably the frontal lobes, extends into the mid-twenties, and is still incomplete long after the end of puberty and linear body growth. There is now abundant evidence that the frequency of behavioral disturbances of adolescence, such as unplanned sexual activity, risk-taking, impulsivity, depression, and delinquency, declines after adolescence despite persistent high levels of gonadal hormones. The most likely explanation for the transient nature of these behavioral disturbances of adolescence is continuing myelination of the frontal cortex and other brain regions that are involved in the executive control of impulses and emotions.

Adolescence is often delayed in foraging societies, resembling our human environments of evolutionary adaptedness. Since the women in these societies have late menarche and are subsequently subfertile, the age of these young women at the time of first birth is 19 years and their husbands are generally several years older. These young parents are strongly supported by older family members, who supply needed food and advice. The mastering of subsistence skills takes many years and an individual generally becomes proficient in these skills in their fourth decade. These realities highlight the adaptive advantages of a post-adolescent or emerging adulthood phase of human maturation, which requires substantial brain maturation and learning.

Secular trends indicate that the duration of pre-adolescent growth and development has shortened over the past two centuries and a further decoupling between pubertal/hormonal maturation and brain maturation has occurred in adolescents in developed societies. The nutritional and social conditions which drive this trend have been previously discussed and reviewed (2). While the mental maturation of adolescents and emerging adults in developed societies is as slow or slower than that of those in predeveloped societies, the onset of puberty in the developed societies now occurs at a younger age than that in the predeveloped societies. Many people in advanced developed states have increasingly recognized the need for prolonged period of education and support beyond adolescence. Others in contrast, especially those in the developing world where traditional structural support systems have collapsed, are often not able to provide the experience of a protected emerging adulthood to their children, leading the United Nations to identify youth, defined as 15–24 years of age, as a demographic group at risk and a special target for intervention (11). The period of emerging adulthood has an evolutionary context and a prolonged maturational underpinning, and we present evidence that supports the idea that emerging adults require protection because they are still both learning *and* maturing. Yet, A literature review and hypotheses of that sort are based on associations. The prolonged dependency and frequent confusion of emerging adults in modern societies is not solely attributable to the complexity of our societies, but also to the fact that they are, intrinsically and physiologically, not yet adults.

## Author Contributions

ZH and MK jointly conceived the article, drafted the manuscript, read, and approved the final version.

### Conflict of Interest

The authors declare that the research was conducted in the absence of any commercial or financial relationships that could be construed as a potential conflict of interest.
